# Open fractures: epidemiological pattern, initial management and challenges in a sub-urban teaching hospital in Nigeria

**DOI:** 10.11604/pamj.2019.33.234.18141

**Published:** 2019-07-19

**Authors:** David Odoyoh Odatuwa-Omagbemi

**Affiliations:** 1 Department of Surgery, Delta State University, Abraka, Nigeria

**Keywords:** Open fractures, management challenges, external fixators, road traffic accidents

## Abstract

**Introduction:**

Management of open fractures poses a constant challenge to Orthopaedic surgeons in Nigeria. Our aim is to determine the epidemiological pattern of open fractures in our centre and share our experiences on the initial management and problems encountered.

**Methods:**

This was an 18 month prospective study of patients that presented with open fractures at our emergency room. Already prepared data collection sheets were used to collect relevant data directly from patients and patients' files.

**Results:**

There were 58 open fractures in 52 patients (31 males and 21 females). Mean age of patients was 36.4 ± 12.2 years. Most patients (82.7%) fell within the age group of 20-49 years. Traders (28.9%) and students (19.6%) were mostly affected. Most open fractures (88.5%) were due to road traffic accidents. The tibia and fibula were the most frequently affected (44.4%). Most injuries were Gustilo *et al.* types IIIA & IIIB (79.3%) open fractures. Patients had initial resuscitation followed by debridement in 42 cases (72%). Fractures were initially stabilized with external fixators in 23 cases (39.7%) and cast slabs in 19 cases (32.8%). The average time between presentation and debridement was 30 hours and average hospital stay was 36 days. Forty two point five per cent of wounds were infected.

**Conclusion:**

Open fractures were mostly due to road traffic accidents and affected the tibia and fibula most frequently with Gustilo *et al.* types IIIA and IIIB forming the bulk of the injuries. Management was challenging with late presentations, scarcity of resources and consequent high rate of infections, prolonged morbidity and hospital stay. These problems were worsened by delay in antibiotic commencement and initial debridement, sub-optimal treatment at peripherial hospitals and mis-management by traditional bone setters.

## Introduction

"An open fracture is one in which a break in the skin allows for direct communication of the fracture site or fracture haematoma with elements external to the usual protection of the skin" [1]. It is estimated that 1 in every 120 persons under the age of 65 years will have fracture and 3% of these fractures are open. Three to six million fractures occur yearly in the United States. Thus fractures are a major public health concern [2, 3]. The degree of soft tissue and bony injuries vary with the amount of energy dissipated during the fracturing process and this eventually also affect the healing process and complication rate. Due to the exposure of the fracture site to the environment and other peculiarities of open fractures, their management poses a serious challenge to every practicing orthopaedic surgeon in spite of recent advances in management protocols. There is increased risk of infections, delayed unions, non-unions and increased amputation rate [4, 5]. The aims of this study are to investigate the epidemiological pattern of open fractures in our centre (Delta State University Teaching Hospital, Oghara, Nigeria), highlight problem areas observed in the process of management and offer suggestions on possible ways forward.

## Methods

This was a prospective study that involved consecutive patients with open fractures that presented at our accident and emergency (A/E) department and were subsequently managed in our centre over a period of 18 months (1^st^ January 2016-30^th^ June 2017). Institutional ethical clearance was gotten after which details of study was explained to each patient and verbal consent obtained by patient agreeing to participate in the study. In addition privacy and confidentially was ensured as the data collection sheet did not provide for names of patients-this was also relayed to patients to reassure them. Already prepared data collection sheets were then used to collect data directly from patients and from records in patients' files by pre-trained registrars in our Orthopaedic unit. Bio-data and data on socio-demographic characteristics of patients were collected in addition to that related to the injuries sustained. Open fractures were classified according to Gustilo *et al.* [6, 7] classification and managed accordingly. Data were analysed using SPSS version 20 and presented here as ratios, means, percentages, tables and charts.

## Results

**Figure 1:**
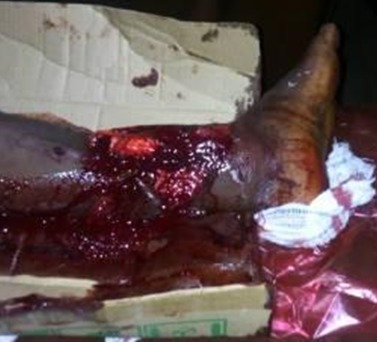
Gustilo and Anderson type III open fracture of right tibia and fibula at presentation

**Figure 2:**
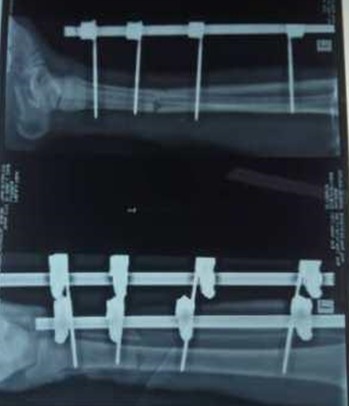
X-ray of the limb after debridement and application of external fixator

Fifty two patients who had 58 open fractures were reviewed during the study period of 18 months. There were 31 males and 21 female giving a male: female ratio of approximately 1.5:1. The mean age of patients was 36.4 ± 12.2 years. Most of the patients (82.7%) fell within the age group of 20-49 years. Traders (28.9%), students (19.6%) and artisans (13.5%) were mostly affected. Most patients (65.5%) were married and 32.7% single. Twenty two patients (42.3%) had secondary education while 17 (32.7%) had tertiary education Table 1. Most open fractures were caused by road traffic accidents in 88.5% of cases, gunshot in 9.6% of cases and assault in 1.9% of cases. The tibia and fibula were the most commonly affected bones (44.4%), followed by the femur (15.5%) Table 2. Most of the injuries (84.4%) were Gustilo *et al* type III open fractures (IIIA = 29.3%; IIIB = 46.6% and IIIC = 8.5%). Gustilo *et al* types I and II formed 3.4% and 12.1% respectively. Patients had initial resuscitation as necessary followed by debridement in 42 cases (72%). Initial fracture stabilization was with external fixators in 23 cases (39.7%) and cast slabs in 19 (cases 32.8%) Table 3. Figure 1 and Figure 2 are clinical photograph and X-ray respectively of a Gustilo type III open fracture of the right tibia and fibula of one our patients. The average time between presentation and debridement was 30 hours and the average hospital stay was 36 days. Forty two point five per cent of wounds were infected. Three of the Gustilo *et al* type IIIC fractures eventually had amputations.

**Table 1:** Socio-demographic characteristic of patients

Age distribution of patients		
Age Group In Years	Frequency	Percentage
10 – 19	2	3.85%
20 – 29	14	26.93%
30 – 39	16	30.77%
40 – 49	13	25.00%
50 – 59	5	9.61%
60 – 69	1	1.92%
70 – 79	1	1.92%
		
**Marital Status**		
Married	34	65.38
Single	17	32.69
Widowed	1	1.92
		
**Educational Status Of Patients**		
Primary	7	13.46%
Secondary	22	42.31%
Tertiary	17	32.69%
None	6	11.54%
		
**Occupation Of Patients**		
Traders	15	28.85%
Schooling	10	19.23%
Artisans	7	13.46%
Unemployed	4	7.69%
Drivers	3	5.77%
Farming	3	5.77%
Civil Servants	3	5.77%
Others	7	13.46

**Table 2:** Initial procedure and methods of stabilization of open fractures

Initial Of Procedure	Frequency	Percentage
Debridement / Irrigation	40	69%
Irrigation Only	5	8.6%
Vascular Repair	2	3.4%
Primary Amputation	3	5.2%
External Fixation	23	39.7%
Pop Slab / Cast	19	32.8%
Skeletal Traction	5	8.6%
K-Wire Fixation	3	5.2%
Tension Band Wiring (Patella)	2	3.4%
Primary Intramedullary Nailing	1	1.7%
Skin Traction	1	1.7%

**Table 3:** Distribution of open fracture-bones affected

Bones Affected	Frequency	Percentage
Tibia/Fibula (Shaft)	26	44.4%
Femur	9	15.5%
Ankle	8	13.8%
Humerus	4	6.9%
Ulna	3	5.2%
Foot Bones	3	5.2%
Patella	3	5.2%
Radius	2	3.4%
Total	58	100%

## Discussion

The management of open fractures has been a challenge since ancient times and remains so to the present day practicing orthopaedic and trauma surgeon and other Physicians who treat fractures [4, 5, 8-10]. There is increased risk of contamination, infection, fracture non-union and mal-unions, delayed union, neurovascular complications, increased amputation rate, prolonged morbidity and mortalities etc, depending on the level of tissue damage [6, 9-12]. The aims of treatment are to prevent infection, ensure early treatment of associated injuries, early soft tissue cover, stabilization of the fracture, achievement of healing and return to work [4, 6, 13]. Open fractures have been reported in the literature to be commoner in males than females [2, 5, 9, 10, 14]. The observation in this study also agrees with this-about 60% of patients in the study being males. This finding is explained by the fact that males are generally more prone to injuries as a result of exposure to risky activities both at work-being largely the bread winners - and at leisure [5, 15]. The average age of patients in this study was 36.4±12.2 years. This average age is much higher than 23±1.5 years reported by Arti *et al* [8], but lower than 38.08 years and 45.5 years recorded by Kombate *et al* [9] and Court-Brown *et al* [10] respectively. However, the last 2 studies were carried out in adult populations of 15 years and above which might partially explain the higher mean age reported. About 83% of patients in this study fell within the ages of 20-49 years.

This is the most economically viable age group in any society. Thus open fractures with their associated morbidity and high cost of treatment do not only consume the scarce resources of low income nations like ours but also reduce productivity by affecting largely the most productive age group. Traders were the most frequently affected by open fractures in our study followed by artisans. Ibeanusi and Ekere [5] from Port Harcourt, Nigeria reported that open tibial fractures were more common in students followed by trader. The fact that these groups of persons are frequently on our roads using public means of transportation such as motorcycles - which are frequently involved in road traffic accidents, might partially be responsible for this observation. Close to 90% of open fractures in this study resulted from road traffic accidents. This is similar to reports by several previous authors [5, 9, 14, 16]. This observation is not surprising considering the fact that road traffic accidents have been reported as the most important cause of trauma generally worldwide with a disproportionate number of such trauma occurring in developing countries [17-19]. The tibia and the fibula bones were the most frequently involved in open fractures making up about 45% of cases in this series. Several previous studies also reported similar findings [9, 16, 20, 21]. The reason for this observation has been ascribed to the fact that the antero-medial border of the tibia is largely subcutaneous throughout its whole length [5, 20]. Gustilo

type III open fractures form the bulk (about 85%) of the open fractures seen in this study with type IIIB accounting for more than half of them. Similarly, Kombate *et al* [9] from Togo reported that 56.3% of open fractures seen in a hospital based study were types III open fractures. In a 15 year review of cases of open fractures, Court-Brown *et al* [10] from the UK in contrast observed, that most of their cases were types II open fractures with type III open fractures accounting for only about 26.8% of their series. This observed difference might be as a result of national health policy variations - these studies being largely hospital based. In the UK for example most open fractures are likely to be managed in designated trauma centres of the type where Court-Brown *et al* [10] did their studies whereas in Africa less severe open fractures are likely to be treated by traditional bone setters (TBS) and some peripherial hospitals with only mostly complicated cases and type III fractures ab initio getting to tertiary health facilities like ours. The average time between injury and presentation to our health facility was 3.32 days ranging from 20 minutes to 28 days. Delayed presentation is a serious challenge in the management of open fractures in our centre as many of the wounds were often frankly infected at presentation with varying degrees of tissue necrosis. This often led to delay in commencing appropriate treatments including initial debridement with consequent poorer outcome of treatment. Reasons given by patients for the delays included: delay in rescue and transportation from injury site to our centre, admission in peripherial hospitals for initial resuscitation before referral and initial treatment by traditional bone setters (TBS).

Another important challenge in the management of open fractures in our centre is the delay in time between presentation and initial debridement. This ranged in this study from 1 hour to 7 days. The major cause of this delay is often patients' inability to pay for treatment. In Nigeria, health insurance coverage is very low and even in those patients that have health insurance, trauma is often not covered. Patients and relations have to pay from their pockets and ours being a poor nation with over 80% of the population leaving below the poverty line, it is often an uphill task to get patients to pay. Our centre operates a "pay before service policy" and patients may not get any form of treatment until they pay. In fact some patients had to be discharged home without treatment or with suboptimal treatment because they could not pay. Those needing more than one procedure in the theatre, may end up with just one. The outcome of treatment of open fractures is poorer as a result of these delays as infection rates and risk of other complications increase. Pollak *et al* [22] observed that there is increased rate of wound infections when debridement is delayed for more than 24 hrs. The delay in presentation, delay in the time of debridement coupled with the delay in antibiotic commencement in addition to suboptimal initial management in peripherial hospitals and the activities of TBS all might in part explain the high wound infection rate of over 42% observed in this study. This infection rate is high compared to that reported in the literature by some previous authors [6, 8, 23]. In addition Gustilo type III fractures formed almost 85% of the open fractures seen in this study. Various reports have also confirmed that infection rates in type III open fractures are far higher than that observed in types I and II open fractures [8, 24-26] which further explains the higher infection rate and morbidity observed in this study. Furthermore, our patients stayed relatively long in the hospital, the average period of stay being about 36 days. This is largely as a result of increased morbidity from infections, the fact that most of the injuries were severe ab initio (Gustilo type III open fractures) and other peculiar sources of delays including scarcity of resources.

## Conclusion

This study has exposed many peculiar problems associated with management of open fractures in our centre with resulting poorer treatment outcomes. The activities of traditional bone setters (TBS) as a source of delay and mismanagement, need to be adequately curtailed through advocacies, public enlightenment on the dangers of patients with open fractures patronising them, legislative control of their activities, training, retraining and certification for them to know their limits and refer cases appropriately. A major root cause of most of the challenges of managing open fractures in our centre is that many of our patients are poor and do not have health insurance coverage. Delay in presentation to hospitals can also be reduced by stationing of ambulances with well trained paramedical staff at intervals on major high ways to quickly extricate, give first aid and transport accident victims to designated hospitals with standard facilities for trauma care.

### What is known about this topic

- The treatment of open fractures remains a challenge to trauma surgeons worldwide in spite of improvements in the level of patients' care;
- Open fractures are commoner in males and young persons worldwide;
- The tibia and the fibula are the bone most frequently affected by open fractures.

### What this study adds

- There is delay in presentation of open fractures to tertiary centres in Nigeria due to the activities of traditional bone setters (TBS), inadequate treatment by less competent peripherial hospitals and ignorance;
- Scarce resources/poverty and delayed presentation of open fractures negatively affect the management of open fractures in our centre resulting in poorer outcomes;
- Gustilo *et al* types III fractures tend to be more common in tertiary hospital based studies in our environment compared with studies from more economically advanced countries.

## Competing interests

The author declares no competing interest.
